# Smoking trends among adolescents from 1990 to 2002 in ten European countries and Canada

**DOI:** 10.1186/1471-2458-6-280

**Published:** 2006-11-10

**Authors:** Anne Hublet, Dirk De Bacquer, Raili Valimaa, Emmanuelle Godeau, Holger Schmid, Giora Rahav, Lea Maes

**Affiliations:** 1 Ghent University, Department of Public Health, De Pintelaan 185, 9000 Ghent, Belgium; 2 University of Jyväskylä, Department of Health Sciences, Research Center for Health Promotion, PO Box 35, 40014 Jyväskylä, Finland; 3 Service Médical du Rectorat de Toulouse, Inserm U558, Association pour le développement d'HBSC, 12 rue Mondran, 31400 Toulouse, France; 4 Swiss Institute for the Prevention of Alcohol and Drug problems, Avenue Ruchonnet 14, 1003 Lausanne, Switzerland; 5 Tel Aviv University, Department of Sociology, School of Social Work, PO Box 39040, 69978 Tel Aviv, Israel

## Abstract

**Background:**

Daily smoking adolescents are a public health problem as they are more likely to become adult smokers and to develop smoking-related health problems later on in their lives.

**Methods:**

The study is part of the four-yearly, cross-national Health Behaviour in School-aged Children study, a school-based survey on a nationally representative sample using a standardised methodology. Data of 4 survey periods are available (1990–2002). Gender-specific daily smoking trends among 14–15 year olds are examined using logistic regressions. Sex ratios are calculated for each survey period and country. Interaction effects between period and gender are examined.

**Results:**

Daily smoking prevalence in boys in 2002 ranges from 5.5% in Sweden to 20.0% in Latvia. Among girls, the daily smoking prevalence in 2002 ranges from 8.9% in Poland to 24.7% in Austria. Three daily smoking trend groups are identified: countries with a declining or stagnating trend, countries with an increasing trend followed by a decreasing trend, and countries with an increasing trend. These trend groups show a geographical pattern, but are not linked to smoking prevalence. Over the 4 surveys, the sex ratio has changed in Belgium, Switzerland, and Latvia.

**Conclusion:**

Among adolescents in Europe, three groups of countries in a different stage of the smoking epidemic curve can be identified, with girls being in an earlier stage than boys. In 2002, large differences in smoking prevalence between the countries have been observed. This predicts a high mortality due to smoking over 20–30 years for some countries, if no policy interventions are taken.

## Background

Daily smoking among adolescents is a significant public health problem. Smoking-related health problems are a function of the duration (years of smoking) and the intensity of use (number of cigarettes smoked) [1]. Most adult smokers began to smoke or were already addicted to nicotine before the age of 18 [2,3]. Besides, a lot of adolescents want to quit smoking, but only a small number of them really succeed [1,2]. Tobacco control policies varied widely in European countries in the last 20 years [4]. The smoking prevalence among adolescents is important for policy makers to monitor their current policy and to make decisions for future policies. Information on recent smoking trends within a country and comparison of trends between countries is therefore urgently needed. This information is important to have a benchmark, in order for countries to see how large their smoking problem is compared with other countries. These data can also help to explain the observed differences and trends in smoking prevalence, by relating it to potentially relevant circumstances in the different countries. Relating smoking trends to country-specific policies regarding smoking, can help policy-makers to determine which actions to take in order to reduce smoking. Studies gathering this information according to a standardised research protocol are rare.

According to the WHO European report on Tobacco Control Policy [5], gender differences in smoking prevalence among young people in Europe are smaller than those for adults. Similarities and differences in smoking trends among boys and girls need consideration for future developments. Here, we present the results of a large international study concentrating on the evolution of daily smoking prevalence among boys and girls between 1990 and 2002. The study targeted 14 and 15 year olds in 10 European countries and Canada.

## Methods

The present paper is based on observations made in the Health Behaviour in School-aged Children study (HBSC). This is a four-yearly cross-national research study conducted in collaboration with the WHO Regional Office for Europe [6]. The data of the 4 last surveys are used (1989–1990, 1993–1994, 1997–1998, and 2001–2002). The HBSC-study is carried out in a growing number of countries (from 16 countries in 1989 to 36 countries in 2001). Only countries participating in the 4 survey periods were included in the analyses: Austria, Belgium, Canada, Finland, Hungary, Latvia, Norway, Poland, United Kingdom (Scotland and Wales), Sweden, and Switzerland. The HBSC study aims to gain insight into young people's health and well-being, health behaviours and their social context. The target population of the study is young people 11, 13 and 15 years old attending school. Cluster sampling (school or classes) is used as sampling method in the study. The survey is carried out on a nationally representative sample in each participating country. The sample consists of more than 1200 students in each year, country and age-category. In this paper, 14-year-old and 15-year-old students were selected (n = 75 745), as daily smoking is still rather rare in younger age groups. More details can be found in the international HBSC protocol [6]. The survey is approved by the Ethics Committee of the University Hospital of Ghent, project 2001/304.

Detailed information on non-response in all countries and all survey years is not available. Non-response at school-level varies between countries and survey years and a decreasing trend can be observed. However, non-response at pupil-level (for this study most important) is more constant between countries and survey years and remains high.

The self-administered questionnaire is completed in the classroom and consists of a standard questionnaire developed by the HBSC international research network. Besides questions on smoking and other health-risk behaviours, there were also questions on health outcomes, individual and social resources... The question used in this paper that remained unchanged over the 4 survey periods, is:

'How often do you smoke tobacco at present?' 'Every day'; 'at least once a week, but not every day'; 'less than once a week'; 'I do not smoke'.

### Statistical analyses

Over the 4 survey periods, prevalence for daily smoking among boys and girls are presented separately. Trends are examined using separate logistic regressions for gender and country. Daily smoking is used as a dependent variable and the survey period as an independent variable, controlling for age. The odds ratios and their 99% confidence interval are computed with reference category 'survey 1990' at one hand (presented in table), and 'survey 2002' on the other hand. An additional analysis focuses on the daily smoking sex ratio (female prevalence of daily smoking/male prevalence of daily smoking), calculated for each survey period and country. Significant differences in this sex ratio are analysed using logistic regressions per country and per survey period, with daily smoking as a dependent variable and gender as an independent variable, controlling for age. The interaction between survey period and gender was also studied using logistic regressions by country and controlling for age. In case it was relevant, the data were weighted with the weights provided by the HBSC national teams [6]. The analyses were done using SPSS 11.0 [7].

## Results

### Daily smoking prevalence in boys

Table 1 shows the daily smoking prevalence classified by survey year and country, for boys and girls separately. The countries are ranked by smoking prevalence in 2002. Among boys, the lowest prevalence in 2002 is found in Sweden, followed by the other participating Western countries, the Eastern European countries and Austria. Looking at the trend from 1990 to 2002, we identified three groups (table 2). Group A includes countries with a significant decline (Finland and Sweden) or stagnation (Norway, Austria and Hungary) in daily smoking over the 4 periods. In group B, Belgium, Canada and the UK show an increase in smoking prevalence in 1994 and 1998, followed by a significant decrease in the last survey of 2002. In Canada and the UK, smoking prevalence in 2002 is not significantly different from the smoking prevalence in 1990. In Belgium however, smoking prevalence in 2002 is still significantly higher than in 1990. Group C includes the Eastern European countries (Poland, Latvia) and Switzerland. Here, smoking prevalence has increased since 1990, followed by a stabilisation in the last survey. The smoking odds between 1990 and 2002 have even been doubled in Latvia and Switzerland.

### Daily smoking prevalence among girls

Among girls, a different pattern concerning smoking prevalence has been observed (table 1). The highest prevalence in 2002 in daily smoking can be found in Austria, Norway and Belgium. The group of countries with the lowest daily smoking prevalence in 2002 includes Eastern European countries (Poland and Latvia) as well as Sweden and Canada. However, in 1990, a clearer geographical pattern is found with the Eastern European countries in the lowest prevalence group, and the Nordic countries in the highest smoking prevalence group. Among girls, the composition of the trend groups is slightly different than among boys. Group A includes Finland, Norway and Sweden where daily smoking prevalence in girls remained constant from 1990 to 2002. In Finland, stabilisation occurred after a decline in 1994 and 1998 compared with 1990. Group B includes the same countries as among boys. But it is remarkable to notice that Canada is the only country in this study where girls have a significantly lower smoking prevalence in 2002 compared with 1990. In group C, daily smoking prevalence increased in 1994 and/or 1998, with a stabilisation between 1998 and 2002 (not in table – odds ratio 1998–2002 (reference): Austria OR = .84; Switzerland OR = 1.18; Latvia OR = 0.77; Poland OR = 1.21, all not significant). An exception is Hungary, where smoking prevalence remained stable till 1998 followed by an increase in 2002. The highest increases in girls' daily smoking prevalence between 1990 and 2002 are found in Latvia (OR 1990 versus 2002 = 8.59) and Switzerland (OR 1990 versus 2002 = 7.38).

### Sex differences in daily smoking prevalence

The sex ratios over the 4 survey periods are presented in table 3. The countries are ranked by sex ratio in 2002. In Sweden and the UK, significantly more girls than boys are smoking daily in 2002. The opposite is true for Latvia and Poland. In the other countries, no significant differences are observed between boys and girls. By studying the significance of the interaction between period and gender, a significant change in sex ratio was observed in 3 countries. In all countries, female smokers caught up with male smokers.

## Discussion

In countries of the European Union with membership before 2004, a converging trend among adult smokers has been observed [8]. However, this trend was not observed in daily smoking among adolescents. Taking into account also some new member states, in 2002 the smoking prevalence among boys varied from 5.5% to 20.0%. Among girls, it varied from 8.9% to 24.7%. It is far from easy to explain this important variation between countries. Policy differences as well as differences in youth cultures can play a role.

Interestingly enough, smoking prevalence within countries is not linked with the observed smoking trends between 1990 and 2002. Among boys as well as girls, three different trends were observed showing the same geographical pattern. Among boys, the Nordic countries show a declining or stabilising smoking trend; in the Western countries an initial increase is followed by a decrease in daily smoking; and in the Eastern European countries an increase is followed by a stabilisation in smoking prevalence between 1998 and 2002. Among girls, similar daily smoking trends can be found, with only a few exceptions. First, no country in this study shows a continuous decline in daily smoking prevalence among girls. Second, Austria and Hungary show an increasing smoking trend in girls, while in boys a stabilisation is observed. Third, Hungary is the only country in this study where smoking prevalence among girls has increased since the last two surveys.

Pirkins et al. [9] state that cross-national data of adolescent substance use should be interpreted cautiously. When comparing data from cross-national surveys, the list of problems includes differences in population focus, differences in sampling method, a different survey context and question wording. The HBSC study attempts to control these problems by adapting standardised methods [6]. Literature on smoking trends using the same methods over different periods and in different countries is very scarce [8].

A weakness in large scale school-based studies is the self-report of substance use. In general, self-reported smoking prevalence has been considered as a good indicator of the actual smoking status, compared with biochemical validated smoking prevalence [10,11], especially in epidemiology. But it may still give an underestimation of the problem in adolescents [11]. Although the questionnaire had to be completed anonymously, cultural differences in answering questions (especially questions with a social stigma) can be a problem (like tobacco use in some countries and certain periods for girls and/or boys). Validation studies in this respect are mostly done in Western countries. It would be interesting to repeat such validation studies in countries with a different cultural background. Another limitation of this school-based study is the fact that school drop outs, which may be a high-risk group for smoking, are not included in the survey (at least in some countries). And finally, information referring to smokeless tobacco is lacking. For instance in Sweden, smokeless tobacco is much used among youngsters, especially boys (14.5% used snuff weekly in 2002) [12]. Hence, in some of the participating countries, the daily smoking prevalence can be an underestimation of the tobacco-related problem in reality.

This paper concentrates only on daily smoking among adolescents, which may give a misleading picture of the whole smoking epidemic. When daily smoking is declining, this behaviour can be overtaken by occasional smoking. According to McNeil [13], smoking among adolescents may well show important fluctuations in regularity, from weekly to daily smoking. However, since daily smoking is defined as an important part of nicotine dependence [14], we decided to use this indicator in order to get a clear picture of the current and future burden of smoking on the public health. Daily smoking adolescents are more likely to smoke in the future and to develop smoking-related health problems leading to premature deaths.

This is a descriptive epidemiological study. To help policy makers, analytical epidemiological studies explaining differences in smoking prevalence and trends are needed. Further analyses are needed on different levels of information (individual, population and country characteristics).

Among adults and, as observed in this study, also among adolescents, gender and country differences in smoking trends follow the four stage model of the smoking epidemic [15], and 'Diffusion of Innovations' theory proposed by Rogers [16]. In the first stage of the smoking epidemic model, smoking begins as a male habit; after men have adopted smoking, females begin to smoke in the second stage; in the third stage, male prevalence begins to decline, while female smoking prevalence remains stable; the fourth phase is characterised by a decline in both genders. It may well be that different countries are facing different stages. However, if this theory holds, most of the countries studied here are found in stage three. This should be further examined. Following Rogers' theory, innovations, such as smoking, are taken up first by communities marked out by their relative advantage in terms of educational level, socioeconomic status and upward social mobility [16]. The observed geographical pattern in smoking trends reflects this theory.

However, these theories do not explain the large differences in smoking prevalence between the countries. As documented in the 2004 ENSP report (European Network for Smoking Prevention), effective tobacco control efforts targeting adolescents are not taken in all countries [4]. Among adolescents, most effects are obtained by increasing taxes and prices, restricting advertising, sponsoring media campaigns and subsidising cessation treatment [4]. Although the whole smoking prevalence pattern cannot be explained by the implementation of these measurements, it is noteworthy that countries scoring high on these components (like the UK, Sweden and Norway) have also a relatively low smoking prevalence, especially among boys. Countries scoring low on these components (like Latvia and Austria) have relatively high smoking prevalence, again especially among boys.

## Conclusion

From this paper, we can conclude that among European adolescents, three groups of countries in a different stage of the smoking epidemic curve can be identified, with girls being in an earlier stage than boys.

As smoking-attributable mortality is most closely related to smoking patterns from thirty or more years earlier and not to the current smoking prevalence [15], the results in this paper predict a huge burden on the health care systems of Eastern European countries over the next 20–30 years. Policy makers in these countries must be encouraged to initiate cost-effective strategies for tobacco control as proposed by the World Bank [17]. But equally important, countries with a declining or stabilising daily smoking trend among adolescents must remain alert. Policy makers there should face the challenge to keep the smoking prevalence declining or at least stable. This can be done by developing initiatives that are innovative and suitable for both boys and girls.

## Competing interests

The author(s) declare that they have no competing interests.

## Authors' contributions

AH: performed the statistical analyses and drafted the manuscript. DDB: gave statistical advice and helped in the interpretation of the results. RV: was involved in revising the manuscript critically. EG: was involved in the international coordination of the study (risk behaviour focus group) and revised the manuscript critically. HS: revised the manuscript critically. GR: revised the manuscript critically. LM: was involved in the design of the study, in drafting the manuscript and revising it critically. All authors have read and approved the final manuscript.

## Pre-publication history

The pre-publication history for this paper can be accessed here:



## Figures and Tables

**Table 1:** Sample characteristics

**Country**	**1990**		**1994**		**1998**		**2002**	
	
	**n**	**% daily smoking**	**n**	**% daily smoking**	**n**	**% daily smoking**	**n**	**% daily smoking**
**Boys**								

Sweden	582	9.5	596	9.7	605	8.6	614	5.5
UK	1739	9.1	1251	13.4	1536	14.5	1249	10.2
Canada	924	9.4	1066	15.0	1177	16.1	592	10.5
Switzerland	629	6.3	658	9.5	930	15.8	754	12.9
Norway	790	17.1	829	15.8	838	17.8	793	15.4
Finland	485	22.7	845	17.3	743	15.7	858	16.4
Belgium	496	10.1	1314	17.8	1089	21.9	1657	16.8
Poland	789	12.7	698	13.8	855	18.5	1010	18.0
Hungary	996	10.7	876	12.7	491	12.8	507	19.1
Austria	546	14.1	1151	20.1	618	20.1	641	19.5
Latvia	346	9.8	501	16.6	573	18.0	530	20.0
**Total**	**8322**	**11.4**	**9785**	**15.3**	**9455**	**16.5**	**9205**	**14.9**

**Girls**								

Poland	692	4.3	705	6.1	782	9.8	1072	8.9
Canada	1016	13.2	1139	19.8	1309	20.4	743	9.0
Switzerland	608	2.1	736	12.3	924	15.8	751	13.0
Latvia	668	1.8	788	5.7	774	9.9	655	13.1
Sweden	541	13.7	562	13.0	541	16.1	606	13.7
UK	1830	11.4	1437	17.5	1604	21.6	1228	16.7
Hungary	1178	7.3	972	9.4	524	6.5	799	16.9
Finland	449	20.3	823	13.9	772	15.7	870	18.0
Belgium	445	13.4	1716	13.9	1113	22.3	1757	19.0
Norway	846	15.7	793	14.9	811	20.5	818	19.9
Austria	478	8.7	717	19.9	755	26.0	631	24.7
**Total**	**8751**	**10.1**	**10388**	**13.8**	**9909**	**17.8**	**9930**	**15.9**

Number of participants and prevalence of daily smoking among 14 and 15 year olds, listed by country and survey period, separately for boys and girls. Countries are listed from lowest daily smoking prevalence to highest daily smoking prevalence in 2002.

**Table 2:** Daily smoking odds ratios (99% confidence interval), 1990 as reference category.

**Country**	**1990**	**1994**	**1998**	**2002**	**Trend group**
**Boys**					

Finland	1	0.72 (0.50–1.04)	0.63 (0.43–0.92)	0.66 (0.46–0.96)*	Group A
Sweden	1	1.00 (0.60–1.66)	0.87 (0.51–1.46)	0.54 (0.30–0.97)*	
Norway	1	0.88 (0.62–1.25)	1.02 (0.73–1.43)	0.85 (0.60–1.22)	
Austria	1	1.54 (0.96–2.47)	1.55 (0.94–2.56)	1.66 (0.99–2.78)	
Hungary	1	0.99 (0.69–1.43)	1.46 (0.93–2.29)	1.37 (0.92–2.06)	
Belgium	1	2.01 (1.31–3.08)**	2.49 (1.61–3.83)**	1.79 (1.18–2.73)**	Group B
Canada	1	1.54 (1.07–2.23)*	1.63 (1.14–2.34)**	1.08 (0.69–1.71)	
UK	1	1.37 (1.00–1.86)	1.51 (1.12–2.02)**	1.06 (0.76–1.47)	
Switzerland	1	1.42 (0.80–2.55)	2.90 (1.76–4.77)**	2.36 (1.40–3.98)**	Group C
Latvia	1	1.78 (1.02–3.13)*	2.11 (1.22–3.65)**	2.20 (1.27–3.79)**	
Poland	1	1.09 (0.73–1.62)	1.58 (1.11–2.27)*	1.55 (1.09–2.20)*	
**Total**	**1**	1.21 (1.08–1.37)**	1.41 (1.26–1.59)**	1.22 (1.08–1.38)**	

**Girls**					

Finland	1	0.67 (0.45–1.01)	0.73 (0.49–1.10)	0.87 (0.60–1.28)	Group A
Sweden	1	0.93 (0.59–1.47)	1.19 (0.76–1.85)	0.99 (0.63–1.54)	
Norway	1	0.93 (0.65–1.37)	1.37 (0.98–1.91)	1.32 (0.95–1.85)	
Belgium	1	1.13 (0.75–1.70)	1.94 (1.28–2.93)**	1.57 (1.05–2.35)	Group B
Canada	1	1.46 (1.07–1.99)*	1.49 (1.10–2.02)*	0.62 (0.41–0.93)*	
UK	1	1.55 (1.19–2.02)**	2.03 (1.58–2.60)**	1.56 (1.18–2.06)**	
Austria	1	2.46 (1.31–4.64)**	3.50 (1.87–6.52)**	4.15 (2.18–7.89)**	Group C
Switzerland	1	5.72 (2.51–13.05)**	8.71 (3.95–19.21)**	7.38 (3.30–16.54)**	
Latvia	1	3.47 (1.48–8.11)**	6.62 (2.93–14.93)**	8.60 (3.83–19.33)**	
Poland	1	1.50 (0.80–2.83)	2.61 (1.47–4.64)**	2.16 (1.24–3.78)**	
Hungary	1	1.14 (0.76–1.73)	1.02 (0.59–1.78)	2.07 (1.41–3.04)**	
**Total**	**1**	1.29 (1.14–1.46)**	1.76 (1.56–1.98)**	1.61 (1.43–1.82)**	

Analyses for boys and girls separately and countries listed by same trend.

**Group A**: declining or stabilising trend; **Group B**: increasing trend followed by decreasing trend; **Group C**: increasing trend with or without stabilisation in last surveys.

* p < 0.01; ** p < 0.001

**Table 3:** Sex ratio of daily smoking.

**Country**	**1990**	**1994**	**1998**	**2002**	**Sex X period (p-value)**
Sweden	1.44	1.34	1.87**	2.49**	.073
UK	1.25	1.30*	1.49**	1.64**	.086
Norway	0.92	0.94	1.15	1.29	.080
Austria	0.62	0.99	1.29	1.27	.016
Belgium	1.33	0.78*	1.02	1.13	.004
Finland	0.89	0.80	1.00	1.10	.263
Switzerland	0.33*	1.29	1.00	1.01	.005
Hungary	0.68*	0.74*	0.51**	0.88	.023
Canada	1.40*	1.32*	1.27*	0.86	.083
Latvia	0.18**	0.34**	0.55**	0.65*	.001
Poland	0.34**	0.44**	0.53**	0.49**	.325
**Total**	0.88*	0.90*	**1.08**	**1.07**	**<.001**

Sex ratio (daily smoking prevalence girls/daily smoking prevalence boys) in the 4 survey periods, gender significance per period and country, controlling for age. P-value of interaction of sex with period, separately for country and controlling for age.

* p < 0.01; ** p < 0.001 (p-value indicating significant differences between boys and girls or to what extent the ratio differs from 1)
